# Ca^2+^-activated mitochondrial biogenesis and functions improve stem cell fate in Rg3-treated human mesenchymal stem cells

**DOI:** 10.1186/s13287-020-01974-3

**Published:** 2020-11-04

**Authors:** Taeui Hong, Moon Young Kim, Dat Da Ly, Su Jung Park, Young Woo Eom, Kyu-Sang Park, Soon Koo Baik

**Affiliations:** 1 Mitohormesis Research Center, Yonsei University Wonju College of Medicine, 20 Ilsan-ro, Wonju, Gangwon-Do 26426 Republic of Korea; 2 Department of Physiology, Yonsei University Wonju College of Medicine, 20 Ilsan-ro, Wonju, Gangwon-Do 26426 Republic of Korea; 3 Department of Internal Medicine, Yonsei University Wonju College of Medicine, 20 Ilsan-ro, Wonju, Gangwon-Do 26426 Republic of Korea; 4 Cell Therapy and Tissue Engineering Center, Yonsei University Wonju College of Medicine, Ilsan-ro 20, Wonju, 26426 Gangwon-Do Republic of Korea; 5 Regeneration Medicine Research Center, Yonsei University Wonju College of Medicine, 20 Ilsan-ro, Wonju, 26426 Gangwon-Do Republic of Korea

**Keywords:** Mesenchymal stem cells, Ginsenoside Rg3, Cellular senescence, Oxidative stress, Mitochondria

## Abstract

Although mitochondrial functions are essential for cell survival, their critical roles in stem cell fate, including proliferation, differentiation, and senescence, remain elusive. Ginsenoside Rg3 exhibits various biological activities and reportedly increases mitochondrial biogenesis and respiration. Herein, we observed that Rg3 increased proliferation and suppressed senescence of human bone marrow-derived mesenchymal stem cells. Osteogenic, but not adipogenic, differentiation was facilitated by Rg3 treatment. Rg3 suppressed reactive oxygen species production and upregulated mitochondrial biogenesis and antioxidant enzymes, including superoxide dismutase. Consistently, Rg3 strongly augmented basal and ATP synthesis-linked respiration with high spare respiratory capacity. Rg3 treatment elevated cytosolic Ca^2+^ concentration contributing to mitochondrial activation. Reduction of intracellular or extracellular Ca^2+^ levels strongly inhibited Rg3-induced activation of mitochondrial respiration and biogenesis. Taken together, Rg3 enhances capabilities of mitochondrial and antioxidant functions mainly through a Ca^2+^-dependent pathway, which improves the proliferation and differentiation potentials and prevents the senescence of human mesenchymal stem cells.

## Introduction

Mitochondria are essential for energy metabolism, calcium homeostasis, cell survival, and apoptosis; however, their role in stem cell proliferation, differentiation, and senescence has not been fully elucidated. In a stem cell niche, quiescent cells have a low mitochondrial mass in a low oxygen environment [1]. These cells have less bioenergetic reliance on oxidative phosphorylation and more glycolytic activity. Furthermore, superoxide production from mitochondrial activities inhibits stemness, negatively affecting stem cell fate [2]. Nevertheless, counteracting evidences demonstrate mitochondrial metabolism exhibits an essential role in stem cell fate and defense against senescence [3]. Active mitochondrial respiration is required to maintain quiescence and the differentiation ability of stem cells [4]. After mitochondria transfer, recipient bone marrow-derived mesenchymal stem cells (BMSCs) displayed increased oxidative phosphorylation, which enhanced proliferation and osteogenic differentiation [5]. Conversely, mitochondrial DNA mutations, lower mitochondrial content, and impaired mitochondrial metabolism lead to stem cell senescence [6].

Rg3 isolated from *Panax ginseng* has various pharmacological actions, including antioxidant [7], anti-inflammatory [8], anti-cancer [9], neuroprotective [10], and anti-aging activities [11]. Rg3 increases insulin sensitivity and inhibits adipogenic differentiation associated with AMP-activated protein kinase (AMPK) activation and peroxisome proliferator-activated receptor γ (*PPARγ*) inhibition, respectively [12, 13]. Rg3 inhibits oxidative stress by elevating catalase and superoxide dismutase (SOD) activities and decreasing xanthine oxidase activity [7]. Additionally, Rg3-related increases in mitochondrial biogenesis and respiration have been reported [14]. However, most of investigation about Rg3 actions had been performed in cancer cells, and little is known about its role in stem cells. In this study, we aimed to resolve the effect of Rg3-induced mitochondrial activation on the fate of human BMSCs.

## Methods

### Cell culture and reagents

Human BMSCs were purchased from American Type Culture Collection. Mononuclear cells were cultured with low-glucose Dulbecco’s modified Eagle’s medium (Gibco) containing 10% fetal bovine serum (Gibco) and 1% penicillin/streptomycin (Gibco) at 37 °C in a 5% CO_2_. Cells with passages 3 or 4 were used for the experiments treated with ginsenoside Rg3 (Cayman Chemical) or 0.25% ethanol as a vehicle. The detailed information is described in Supplemental Methods.

### Quantitative polymerase chain reaction (qPCR)

Total RNA was isolated from BMSCs and used to synthesize cDNA. Transcript levels were measured by QuantStudio™ 6 Real-Time PCR System (Applied Biosystems) using sequence-specific primers listed in supplementary figure 1. The cycle threshold (Ct) values of the target genes were normalized to control (peptidylprolyl isomerase A), and the relative amount was calculated by using the equation, 2^−ΔΔCt^.

### Western blot analysis

The cells were lysed using RIPA lysis buffer, and lysates were separated by 10–15% sodium dodecyl sulfate-polyacrylamide gel electrophoresis and transferred to polyvinylidene fluoride membranes. The membranes were incubated overnight with primary antibodies, the detailed information of which is described in Supplemental Methods. After washing, the membranes were incubated for 1 h in horseradish peroxidase-conjugated secondary antibodies. The membranes were incubated in a developing solution (GE Healthcare), and signal was detected using Chemi-Doc System (Bio-rad).

### Senescence-associated β-galactosidase (SA-β-gal) staining

SA-β-gal activity was assessed with a senescence β-gal staining kit (Cell Signaling Technologies) according to the manufacturer’s instructions.

### Adipogenic and osteogenic differentiation

After incubation with vehicle or Rg3 for 5 days, BMSCs were maintained for 2 or 3 weeks in either osteogenic or adipogenic medium. The osteogenic medium contains 10 mM β-glycerophosphate (Sigma), 0.2 mM ascorbic acid-2-phosphate (Sigma), and 100 nM dexamethasone (Sigma). After 14 days, Alizarin Red staining was performed and the absorbance at 540 nm was detected by spectrophotometer. The adipogenic medium contains 1 mM dexamethasone, 0.5 mM 1-methyl-3-isobutylxanthine (Sigma), 100 μM indomethacin (Sigma), and 10 μg/mL insulin (Sigma). After 21 days, Oil Red O staining was performed and the absorbance at 520 nm was detected.

### Oxygen consumption rate (OCR) and extracellular acidification rate (ECAR) measurement

OCR and ECAR were measured in BMSCs using the Seahorse XFe96 Analyzer (Agilent Technologies) in response to XF Cell Mito Stress Test Kit or Glycolysis Stress Test Kit (Agilent Technologies). Supplemental Experimental Procedures provides detailed information about OCR and ECAR measurement from BMSCs.

### Cytosolic ROS measurement

BMSCs were loaded with 5 μM DCFH_2_DA (Invitrogen) for 20 min at 37 °C, and fluorescence intensity reflecting ROS production was measured at the excitation and emission wavelengths of 485 and 538 nm using a fluorescence-activated cell sorting (FACSAria III, BD Biosciences, USA).

### Live-cell Ca^2+^ imaging

BMSCs seeded on 12-mm coverslip were loaded with 5 μM Fura-2/AM (Thermo Fisher Scientific) for 30 min at 37 °C. Then, cells were transferred to a perfusion chamber on an inverted microscope (IX73, Olympus). Fluorescence images with an illuminator (pe-340Fura; CoolLED) were captured at 510 nm with a CCD camera (Prime-BSI; Teledyne Photometrics), and the ratio of fluorescence intensities (F_340_/F_380_) reflecting intracellular Ca^2+^ was analyzed by MetaFluor 6.1 (Molecular Devices).

### Statistical analysis

Student’s *t* test was used to compare means of two data groups. One-way ANOVA was used to compare means of two or more data groups. All graphs and statistical analysis were performed using software (Prism version 6.0, GraphPad Software). Data are presented as means ± SEMs, and *P* < 0.05 was considered significant.

## Results

### Rg3 augments proliferation and reduces senescence in human BMSCs

**Fig. 1:**
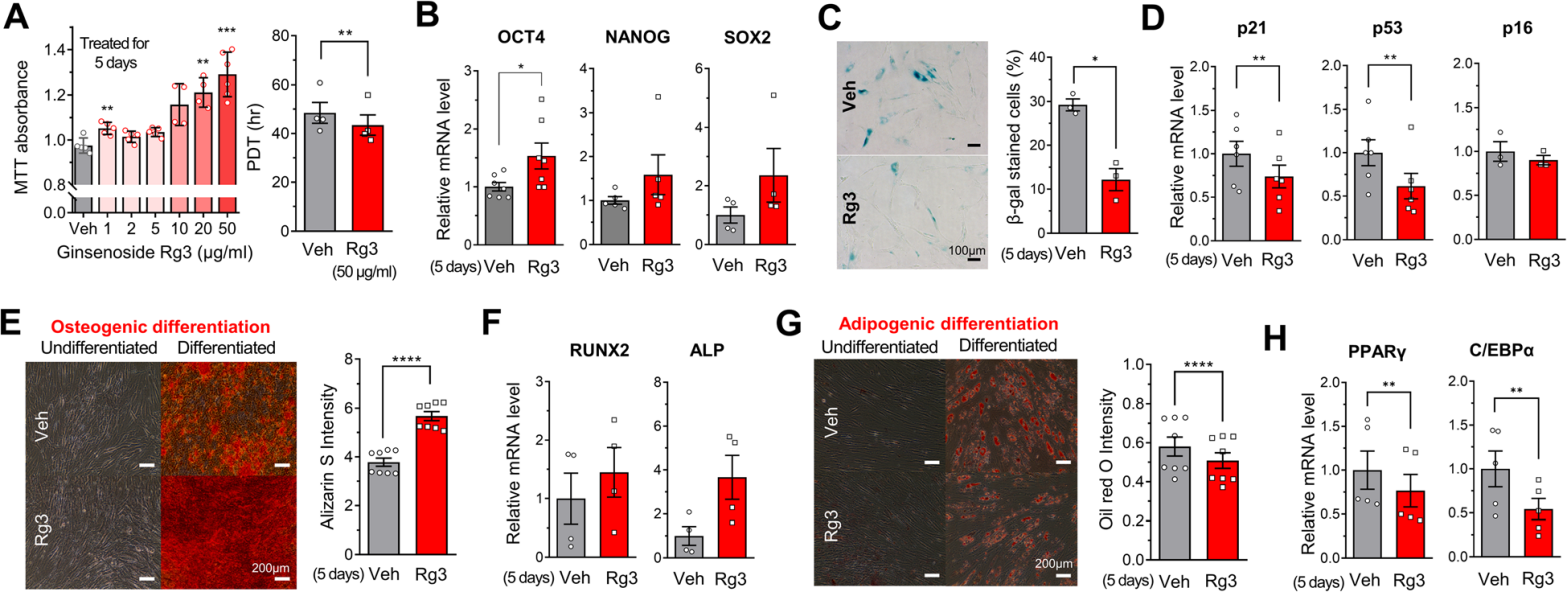
Rg3 augments proliferation and reduces senescence in human BMSCs. **a** Increased proliferation of human BMSCs by Rg3 was estimated via the MTT absorbance and proliferation doubling time (PDT). **b** Transcriptional levels of stemness genes, *OCT4*, *NANOG*, and *SOX2*, were evaluated using quantitative real-time PCR. Attenuation of cellular senescence was estimated by the **c** percentage of β-galactosidase staining positive cells and **d** expression of senescence markers, *p21*, *p53*, and *p16*. Osteogenic differentiation was quantified via **e** the absorbance (optical density) of Alizarin Red S staining and **f** expression of osteogenic genes, *RUNX2* and *ALP*. Adipogenic differentiation was quantified via **g** the absorbance of Oil Red O staining and **h** expression of lipogenic genes, *PPARγ* and *C/EBPα*. Data are presented as the means ± SEMs; *P < 0.05, **P < 0.01, ***P < 0.001, and ****P < 0.0001

Human BMSCs exposed to Rg3 for 5 days showed increased proliferation in a dose-dependent manner, with a shortened population doubling time (Fig. 1a). The expression of stemness genes, including *OCT4*, *NANOG*, and *SOX2*, tends to be increased by Rg3 treatment (Fig. 1b). As an indicator of senescence, β-galactosidase staining showed less positive (senescent) cells in the Rg3-treated cells than in the control cells (Fig. 1c). Rg3 treatment downregulated the transcriptional levels of *p21* and *p53*, which are important molecules involved in cell cycle arrest and senescence (Fig. 1d). To investigate differentiation potency, BMSCs were treated with vehicle or Rg3 for 5 days and then cultured in osteogenic and adipogenic differentiation media for 14 and 21 days, respectively. Rg3 increased Ca^2+^ deposition, as measured by Alizarin Red S staining (Fig. 1e). Osteogenic genes such as RUNX family transcription factor 2 (*RUNX2*) and alkaline phosphatase (*ALP*) were upregulated by Rg3 (Fig. 1f). Conversely, adipogenic differentiation, detected via Oil Red O staining, was attenuated by Rg3 (Fig. 1g). The expression of the adipogenic genes, *PPARγ* and CCAAT-enhancer-binding protein α (*C/EBPα*), was lower in Rg3-treated cells (Fig. 1h).

### Rg3 reduces oxidative stress and increases mitochondrial biogenesis and metabolism

**Fig. 2:**
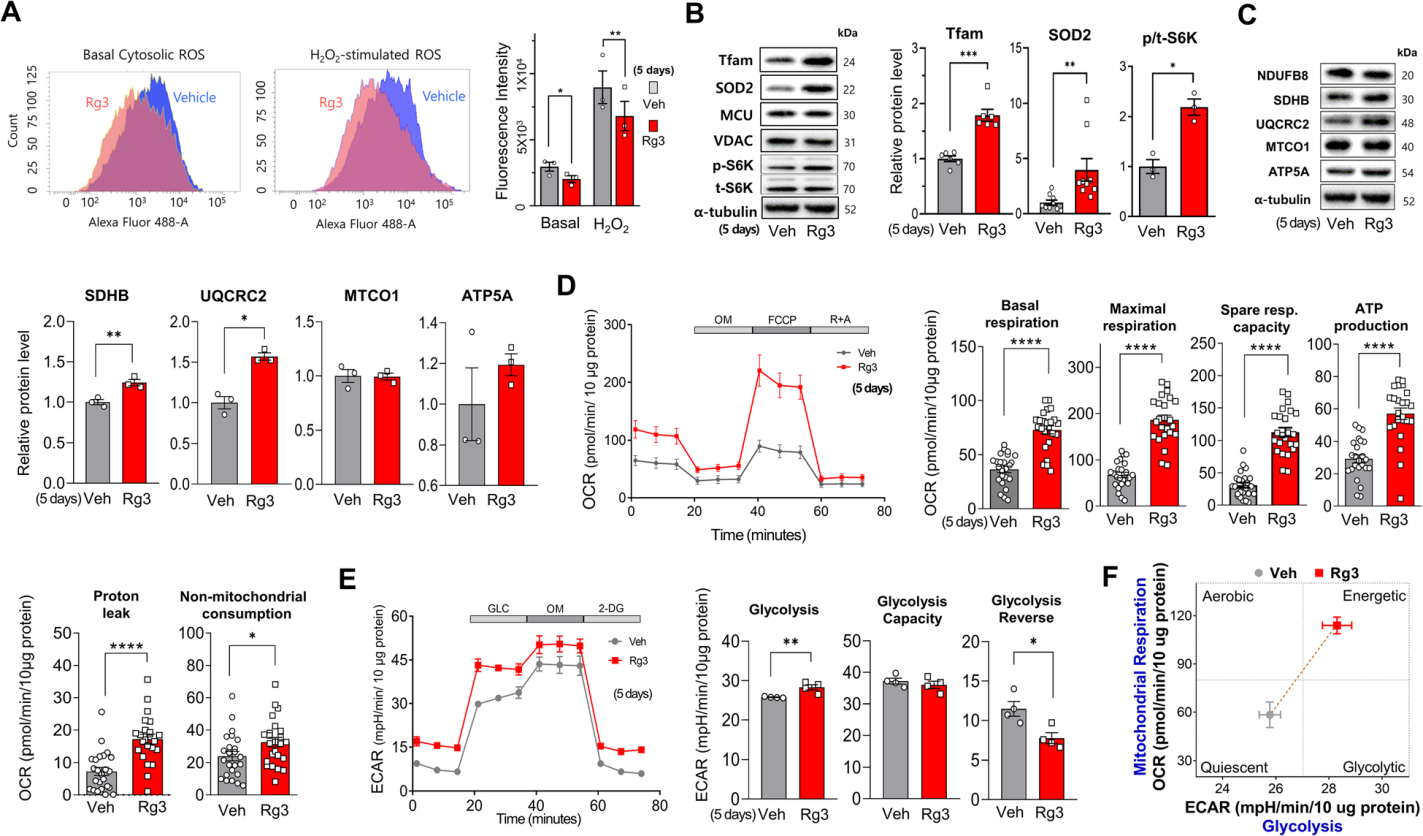
Rg3 reduces oxidative stress and increases mitochondrial biogenesis and metabolism. **a** Reactive oxygen species (ROS) production under basal and H_2_O_2_-treated conditions was measured using flow cytometry with DCF-DA staining. **b**, **c** Alterations in protein levels of mitochondrial transcription factor (Tfam), superoxide dismutase 2 (SOD2), phosphorylated p70S6 kinase (S6K), and electron transport chain proteins (NDUFB8, SDHB, UQCRC2, MTCO1, ATP5A). **d**–**f** Mitochondrial oxygen consumption rate (OCR; **d**) and extracellular acidification rate (ECAR; **e**) were measured using the Seahorse XFe96 metabolic flux analyzer and **f** the energy map of ECAR and OCR values was constructed. Data are presented as the means ± SEMs; *P < 0.05, **P < 0.01, ***P < 0.001, and ****P < 0.0001

Reactive oxygen species (ROS) production, known to cause stem cell senescence, was reduced by 5 days of Rg3 treatment, both under basal and increased oxidative stress conditions (Fig. 2a). The expression of antioxidant enzymes, including mitochondrial SOD (SOD2) and catalase, was increased by Rg3 (Fig. 2b and Fig. S1A). As upstream regulators of mitochondrial biogenesis, PPARγ coactivator 1α (PGC1α) and mitochondrial transcription factor A (Tfam) were also significantly upregulated upon Rg3 exposure. The p70S6 kinase (S6K), a downstream mammalian target of rapamycin (mTOR), was activated by Rg3. Components of the respiratory chain, including succinate dehydrogenase B (SDHB) and ubiquinol-cytochrome c reductase complex 2 (UQCRC2), were upregulated by Rg3 (Fig. 2c). Mitochondrial oxygen consumption by BMSCs was markedly increased following 5 days of Rg3 treatment, as was basal and maximal respiration, ATP-linked consumption, spare capacity, and proton leak (Fig. 2d). Glycolytic activity was also augmented, and the glycolysis reserve was diminished by Rg3 (Fig. 2e). Activation of mitochondrial respiration and glycolysis by Rg3 shifted the metabolic phenotype of BMSCs from a quiescent to an energetic state (Fig. 2f).

### Rg3 induces mitochondrial biogenesis and activation mediated by Ca^2+^ increase

**Fig. 3:**
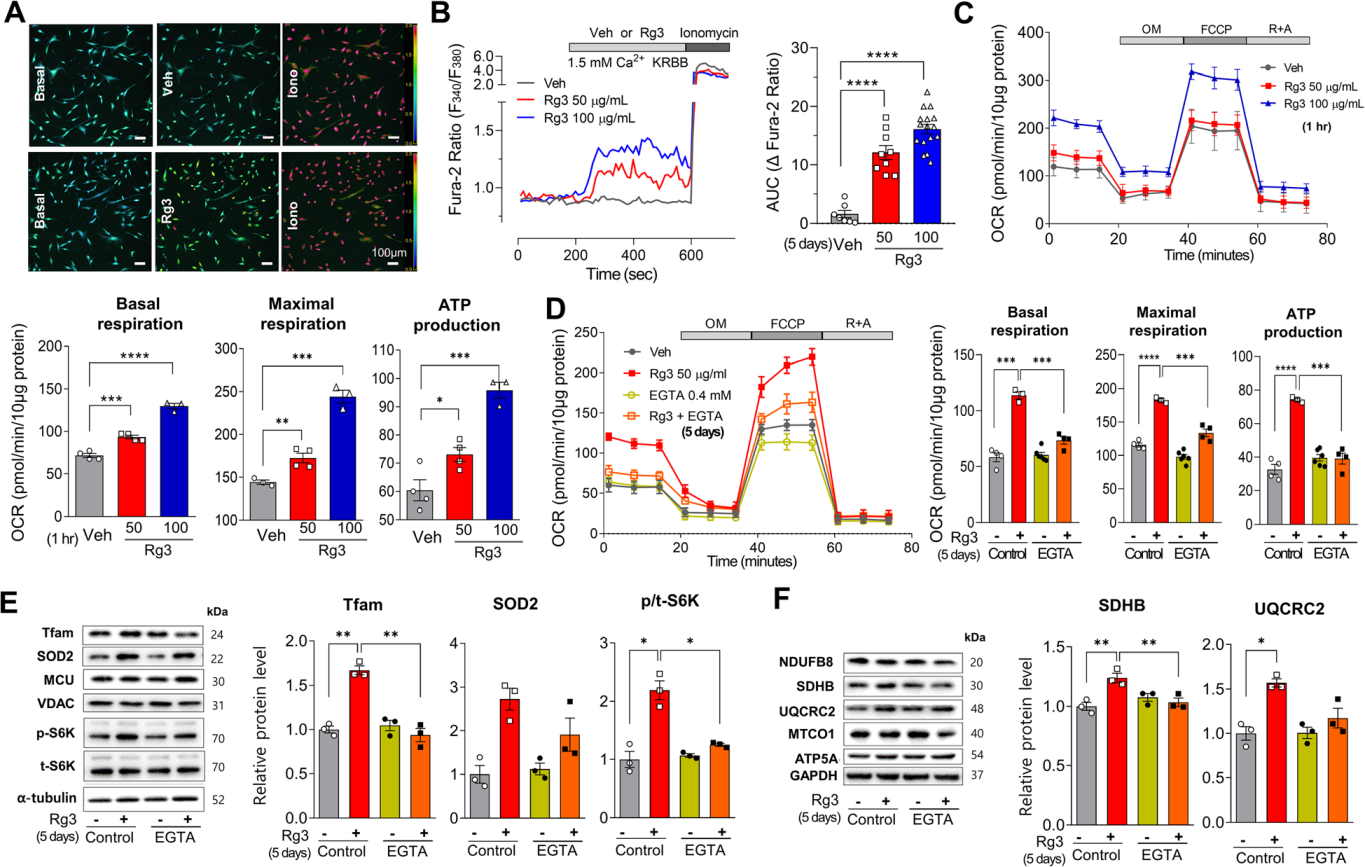
Rg3 induces mitochondrial biogenesis and activation mediated by Ca^2+^ increase. **a** [Ca^2+^]_*i*_ measurement using a fluorescence imaging system after Fura-2-AM dye loading. **b** Rg3 induced sustained [Ca^2+^]_*i*_ elevation, as estimated by the area under the curve (AUC) above the baseline. **c** Mitochondrial oxygen consumption rate (OCR) was dose-dependently increased by 1-h incubation of Rg3. **d**–**f** Effects of extracellular Ca^2+^ reduction by the addition of 0.4 mM EGTA on OCR (**d**), and protein levels of mitochondrial transcription factor (Tfam), phosphorylated p70S6 kinase (S6K), and electron transport chain complex II (SDHB) (**e**, **f**) following 5 days of Rg3 incubation. Data are presented as the means ± SEMs; *P < 0.05, **P < 0.01, ***P < 0.001, and ****P < 0.0001

To identify the molecular mechanism underlying mitochondrial biogenesis and functional activation, we performed a real-time measurement of intracellular Ca^2+^ concentration ([Ca^2+^]_*i*_) in BMSCs loaded with Fura-2 (Fig. 3a). Acute exposure to Rg3 increased [Ca^2+^]_*i*_ under either 1.5 mM extracellular Ca^2+^ (Fig. 3b) or Ca^2+^ free (Fig. S2A) condition. Mitochondrial respiration was dose-dependently increased by Rg3 over 1 h treatment (Fig. 3c). Rg3 activated AMPK and cAMP response element-binding protein (CREB), which are involved in Ca^2+^ activated signaling (Fig. S1B and S1C). Mitochondrial biogenesis and activation by acute (1 h) and chronic (5 days) Rg3 incubation were abrogated by intracellular Ca^2+^ chelation (Fig. S2B) or by reducing extracellular Ca^2+^ with 0.4 mM EGTA (Fig. 3d), respectively. Upregulation of mitochondrial proteins, including Tfam and SDHB, by Rg3 was abolished by extracellular EGTA addition (Fig. 3e, f).

## Discussion

Mitochondria are important bioenergetic organelles, but functional consequences of mitochondrial activation in stem cells have been underestimated. Recent advances have shown that adequate mitochondrial function in stem cells is essential to maintain proliferation and differentiation abilities [2, 3]. Inhibition of mitochondrial fatty acid oxidation or impaired clearance of dysfunctional mitochondria deteriorates stem cell function and fate [15, 16]. Here, we demonstrated that upregulated mitochondrial biogenesis and respiratory capacity by ginsenosides Rg3 are related to improved stem cell fates, including increased proliferation, reduced senescence, and facilitated osteogenic differentiation of human BMSCs. Mitochondrial activation via increased [Ca^2+^]_*i*_ by Rg3 leads to energetic and healthy mitochondria with reduced oxidative stress. Overall, [Ca^2+^]_*i*_-mediated activation of mitochondrial metabolism and the antioxidant system improves stem cell fate in human BMSCs.

Previous reports on mitochondrial actions of Rg3 or ginseng extracts in myoblast cells are consistent with our findings in stem cells [14, 17]. Furthermore, Rg3 treatment protected lipopolysaccharide-induced mitochondrial dysfunction in hepatocytes by increasing mitochondrial biogenesis and functions via AMPK signaling [18]. We observed that Rg3 increased [Ca^2+^]_*i*_ by intracellular Ca^2+^ release and extracellular Ca^2+^ influx. This [Ca^2+^]_*i*_ rise was responsible for early and late increases in basal, maximal, and ATP synthesis-linked respiration. Rg3 activated AMPK and CREB at different time points, both of which are involved in Ca^2+^-activated upregulation of PGC1α and mitochondrial biogenesis. Additionally, mitochondrial Ca^2+^ uptake increases activities of matrix dehydrogenases, oxygen consumption, and ATP synthesis [19]. Therefore, it is conceivable that increased mitochondrial respiration could be attributed to cytosolic and mitochondrial Ca^2+^ rises and their consequences on mitochondrial biogenesis.

Accumulating evidence suggests that oxidative stress in stem cells accelerates senescence, inhibits proliferation, and enhances adipogenic but reduces osteogenic differentiation [20, 21]. We demonstrated that Rg3 suppressed cytosolic ROS levels under both basal and H_2_O_2_-treated conditions. Antioxidant defense mechanisms were reinforced by Rg3 incubation, as observed by increased SOD2 and catalase, which is compatible with the result of ROS measurement. While Rg3-induced increases in SOD activity have been previously reported, the molecular mechanisms underlying this response have not been fully elucidated [22]. It has been shown that PGC1α activated antioxidant genes, including SOD and catalase [23, 24]. We suggest that Rg3 increases antioxidant enzyme expression with mitochondrial biogenesis, thereby attenuating senescence by suppressing oxidative stress.

In conclusion, Rg3 increases mitochondrial biogenesis and activities along with antioxidant effects, which are associated with improved stem cell functions in BMSCs. Of note, Rg3 induced intracellular Ca^2+^ elevation, mediating Rg3-induced mitochondrial upregulation. We observed that Rg3 increases mitochondrial fitness, as estimated by a bioenergetic health index (Fig. S2C), which indicates efficient ATP synthesis and ample reserve capacity [25]. While further investigations are necessary to demonstrate the molecular mechanisms underlying the causal relationship between mitochondrial metabolism and stemness maintenance in BMSCs, we nonetheless suggest that mitochondrial activation via appropriate Ca^2+^ elevation could be an effective strategy for stem cell maintenance and differentiation, aimed at increasing the number of healthy mitochondria and inducing a stronger defense against oxidative stress. The therapeutic preservation of well-functioning mitochondria and a robust antioxidant defense could benefit stem cell transplantation, as well as slow the aging process of endogenous stem cells.

## Supplementary information

Additional file 1: **Supplementary Fig. S1.** Rg3 activates cell signaling and mitochondrial biogenesis**.** (A) Transcriptional regulations of main regulator of mitochondrial biogenesis (PGC1α) and antioxidant enzymes (SOD2 and catalase) by Rg3 were measured by quantitative PCR. (B, C) Effects of acute (1 h; B) and chronic (5 days; C) exposure of Rg3 on AMP-activated protein kinase (AMPK) and cAMP response element-binding protein (CREB) activation. Data are presented as means ± SEMs, * *P* < 0.05 and ** *P* < 0.01. **Supplementary Fig. S2**. Ca^2+^ increase by Rg3 activates mitochondrial respiration. (A) Increased cytosolic Ca^2+^ concentration ([Ca^2+^]_*i*_) under extracellular Ca^2+^ free condition. [Ca^2+^]_*i*_ measurement using fluorescence imaging system after Fura-2-AM dye loading. Rg3-induced [Ca^2+^]_*i*_ elevations under Ca^2+^ free and normal Ca^2+^ conditions were compared by the area under the curve (AUC) above the baseline. (B) Role of intracellular Ca^2+^ increase on acute Rg3-induced mitochondrial activation. BAPTA-AM (10 or 20 μM) were pretreated for chelating intracellular Ca^2+^. Effects of intracellular Ca^2+^ reduction on mitochondrial oxygen consumption rate (OCR) changes by 1 h exposure of Rg3 were evaluated. (C) Rg3-induced augmentation of biological health index. Based on oxygen consumption rate (OCR) data in Fig. 2D, enhanced biological health index by Rg3 was calculated by the equation as follow; BHI = log_10_[(ATP synthesis-linked) *×* (Spare Respiratory capacity) / (Proton leak) *×* (Non-mitochondrial respiration)]. Data are presented as means ± SEMs, * *P* < 0.05, ** *P* < 0.01, *** *P* < 0.001 and **** *P* < 0.0001. **Supplementary Fig. S3**. Role of sustained Ca^2+^ elevation by Rg3 on stem cell fates. (DOCX 3166 kb)

## Data Availability

The authors agree to share data and materials related to this manuscript.

## Abbreviations

ALP: Alkaline phosphatase
AMPK: AMP-activated protein kinase
BMSCs: Bone marrow-derived mesenchymal stem cells
[Ca^2+^]_*i*_: Intracellular Ca^2+^ concentration
C/EBPα: CCAAT-enhancer-binding protein α
CREB: cAMP response element-binding protein
PGC1α: PPARγ coactivator 1α
PPARγ: Peroxisome proliferator-activated receptor γ
ROS: Reactive oxygen species
RUNX2: RUNX family transcription factor 2
SDHB: Succinate dehydrogenase B
SOD: Superoxide dismutase
S6K: p70S6 kinase
Tfam: Mitochondrial transcription factor A
UQCRC2: Ubiquinol-cytochrome c reductase complex 2
