# Supplementary material for: Ca2+-activated mitochondrial biogenesis and functions improve stem cell fate in Rg3-treated human mesenchymal stem cells
Source: Stem Cell Res Ther. 2020 Nov 4;11:467. doi: 10.1186/s13287-020-01974-3 (PMC7640456; doi:10.1186/s13287-020-01974-3)
Supplement: Supplementary file 1 — Supplementary Fig. S1. Rg3 activates cell signaling and mitochondrial biogenesis. (A) Transcriptional regulations of main regulator of mitochondrial biogenesis (PGC1α) and antioxidant enzymes (SOD2 and catalase) by Rg3 were measured by quantitative PCR. (B, C) Effects of acute (1 h; B) and chronic (5 days; C) exposure of Rg3 on AMP-activated protein kinase (AMPK) and cAMP response element-binding protein (CREB) activation. Data are presented as means ± SEMs, * P < 0.05 and ** P < 0.01. Supplementary Fig. S2. Ca2+ increase by Rg3 activates mitochondrial respiration. (A) Increased cytosolic Ca2+ concentration ([Ca2+]i) under extracellular Ca2+ free condition. [Ca2+]i measurement using fluorescence imaging system after Fura-2-AM dye loading. Rg3-induced [Ca2+]i elevations under Ca2+ free and normal Ca2+ conditions were compared by the area under the curve (AUC) above the baseline. (B) Role of intracellular Ca2+ increase on acute Rg3-induced mitochondrial activation. BAPTA-AM (10 or 20 μM) were pretreated for chelating intracellular Ca2+. Effects of intracellular Ca2+ reduction on mitochondrial oxygen consumption rate (OCR) changes by 1 h exposure of Rg3 were evaluated. (C) Rg3-induced augmentation of biological health index. Based on oxygen consumption rate (OCR) data in Fig. 2D, enhanced biological health index by Rg3 was calculated by the equation as follow; BHI = log10[(ATP synthesis-linked) × (Spare Respiratory capacity) / (Proton leak) × (Non-mitochondrial respiration)]. Data are presented as means ± SEMs, * P < 0.05, ** P < 0.01, *** P < 0.001 and **** P < 0.0001. Supplementary Fig. S3. Role of sustained Ca2+ elevation by Rg3 on stem cell fates. (DOCX 3166 kb) [file 13287_2020_1974_MOESM1_ESM.docx]

**Supplementary Figure Legends**

**Supplementary Figure S1. Rg3 activates cell signaling and mitochondrial biogenesis.** (A) Transcriptional regulations of main regulator of mitochondrial biogenesis (PGC1α) and antioxidant enzymes (SOD2 and catalase) by Rg3 were measured by quantitative PCR. (B, C) Effects of acute (1 h; B) and chronic (5 days; C) exposure of Rg3 on AMP-activated protein kinase (AMPK) and cAMP response element-binding protein (CREB) activation. Data are presented as means ± SEMs, * *P*<0.05 and ** *P*<0.01.

**Supplementary Figure S2. Ca^2+^ increase by Rg3 activates mitochondrial respiration.** (A) Increased cytosolic Ca^2+^ concentration ([Ca^2+^]*_i_*) under extracellular Ca^2+^ free condition. [Ca^2+^]*_i_* measurement using fluorescence imaging system after Fura-2-AM dye loading. Rg3-induced [Ca^2+^]*_i_* elevations under Ca^2+^ free and normal Ca^2+^ conditions were compared by the area under the curve (AUC) above the baseline. (B) Role of intracellular Ca^2+^ increase on acute Rg3-induced mitochondrial activation. BAPTA-AM (10 or 20 μM) were pretreated for chelating intracellular Ca^2+^. Effects of intracellular Ca^2+^ reduction on mitochondrial oxygen consumption rate (OCR) changes by 1h exposure of Rg3 were evaluated. (C) Rg3-induced augmentation of biological health index. Based on oxygen consumption rate (OCR) data in Fig. 2D, enhanced biological health index by Rg3 was calculated by the equation as follow; BHI = log_10_[(ATP synthesis-linked) *×* (Spare Respiratory capacity) / (Proton leak) *×* (Non-mitochondrial respiration)]. Data are presented as means ± SEMs, * *P*<0.05, ** *P*<0.01, *** *P*<0.001 and **** *P*<0.0001.

**Supplementary Figure S3. Role of sustained Ca^2+^ elevation by Rg3 on stem cell fates.**

**
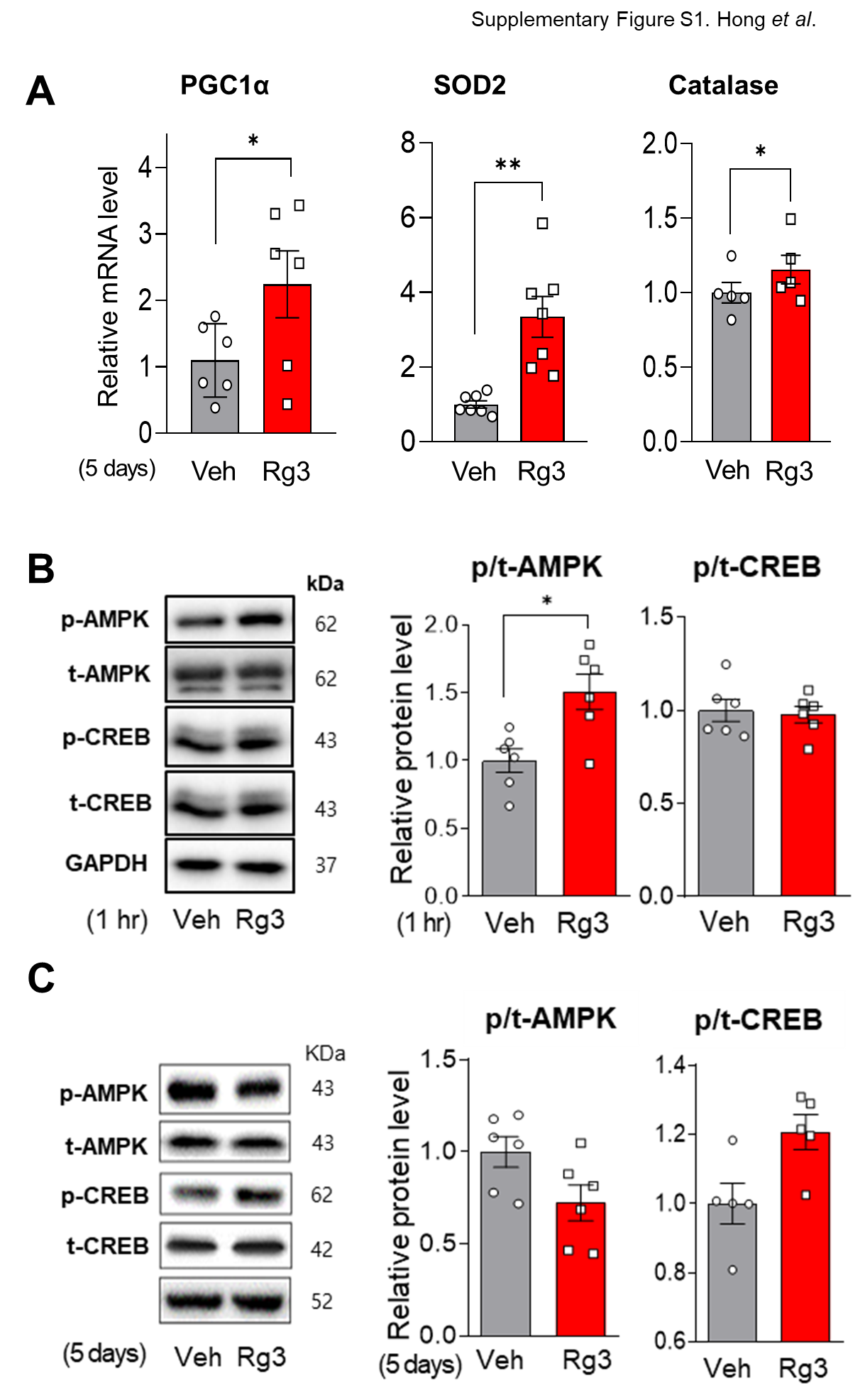
**

**
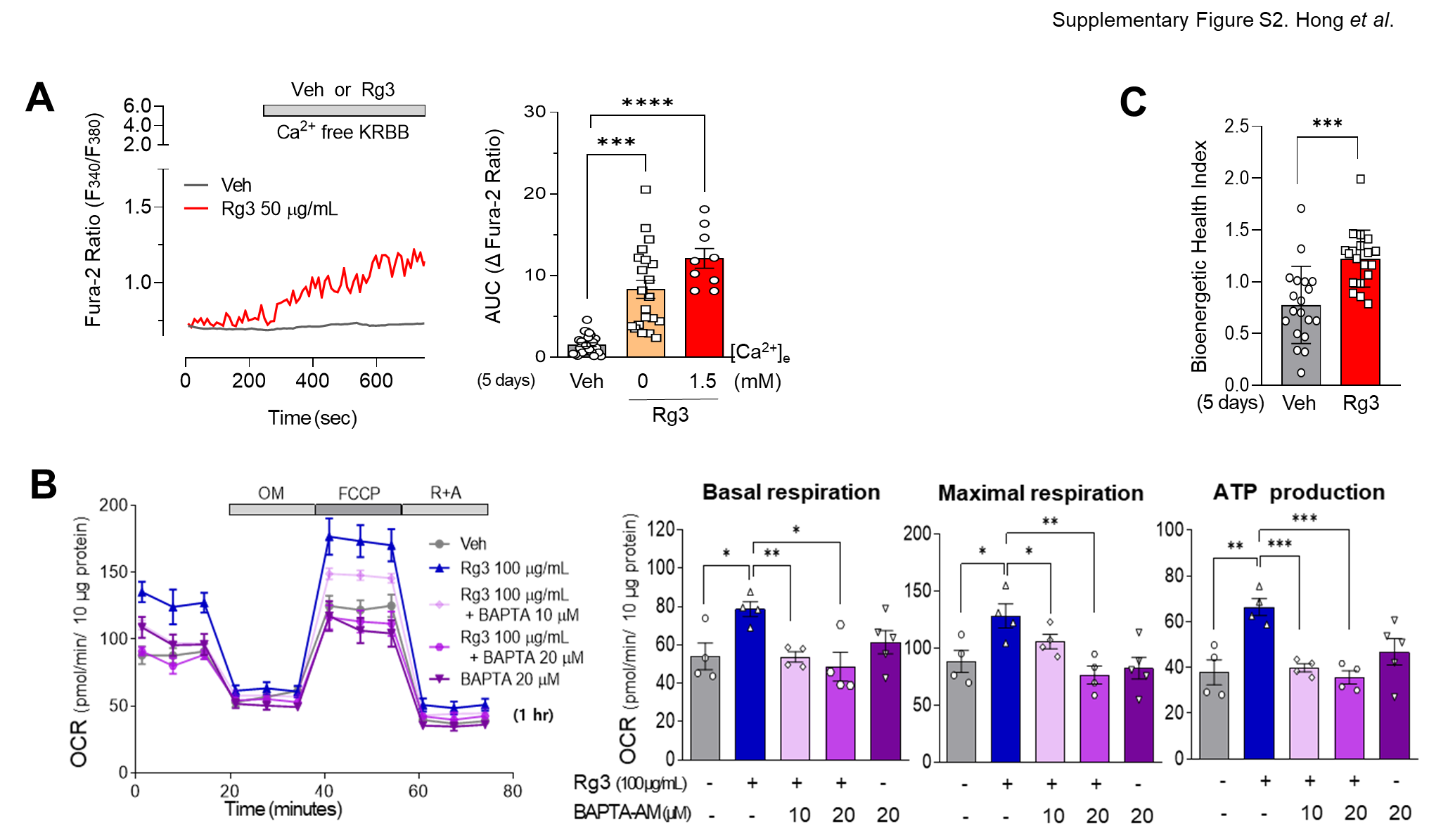
**

**
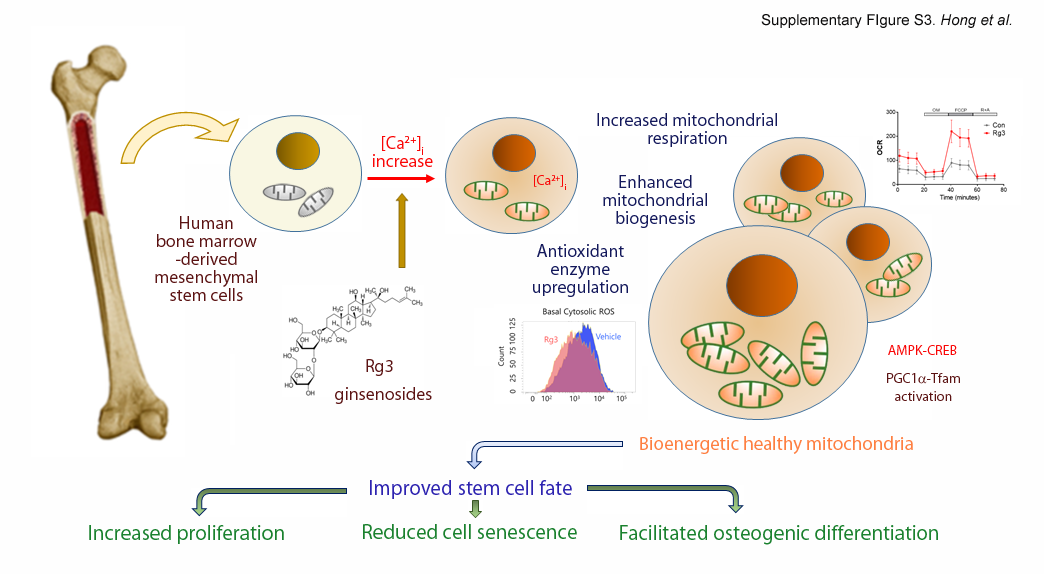
**

**Supplementary table 1. List of primer sequence in quantitative PCR**

| Gene |  | Primer sequence |
| --- | --- | --- |
| OCT4 | Forward | 5'- ACATCAAAGCTCTGCAGAAAGAACT -3' |
|  | Reverse | 5'- CTGAATACCTTCCCAAATAGAACCC -3' |
| SOX2 | Forward | 5'- GGGAAATGGGAGGGGTGCAAAAGAGG -3' |
|  | Reverse | 5'- TTGCGTGAGTGTGGATGGGATTGGTG -3' |
| NANOG | Forward | 5'- ACCTATGCCTGTGATTTGTGG -3' |
|  | Reverse | 5'- AGTGGGTTGTTTGCCTTTGG -3' |
| p21 | Forward | 5'- AGCAGCGGAACAAGGAGT -3' |
|  | Reverse | 5'- TTACAGTCTAGGTGGAGAAACG -3' |
| p53 | Forward | 5'- GAGGTTGGCTCTGACTGTACC -3' |
|  | Reverse | 5'- TCCGTCCCAGTAGATTACCAC -3' |
| p16 | Forward | 5'- TTCCCCCACTACCGTAAATGT -3' |
|  | Reverse | 5'- GCTCACTCCAGAAAACTCCAAC -3' |
| ALP | Forward | 5'- CACCCACGTCGATTGCATCT -3' |
|  | Reverse | 5'- TAGCCACGTTGGTGTTGAGC -3' |
| RUNX2 | Forward | 5'- GTGGACGAGGCAAGAGTTTCA -3' |
|  | Reverse | 5'- CATCAAGCTTCTGTCTGTGCC -3' |
| PPARG | Forward | 5'- AGCCTCATGAAGAGCCTTCCAAC -3' |
|  | Reverse | 5'- TCTCCGGAAGAAACCCTTGCATC -3' |
| CEBPA | Forward | 5'- TGTATACCCCTGGTGGGAGA -3' |
|  | Reverse | 5'- TCATAACTCCGGTCCCTCTG -3' |
| PGC1A | Forward | 5'- TCCTTTCTCTCGCCCAACACGATCT -3' |
|  | Reverse | 5'- GCATCCGACAGGACAAACAGTGGA -3' |
| SOD2 | Forward | 5'- TGGTGGTCATATCAATCATAGC -3' |
|  | Reverse | 5'- ATTTGTAAGTGTCCCCGTTC -3' |
| CAT | Forward | 5’- TGGGATCTCGTTGGAAATAACAC -3’ |
|  | Reverse | 5’- TCAGGACGTAGGCTCCAGAAG -3’ |
| PPIA | Forward | 5'- TCCTGGCATCTTGTCCAT -3' |
|  | Reverse | 5'- TGCTGGTCTTGCCATTCCT -3' |

**Supplemental Methods**

**1. Cell culture and reagents**

Human bone marrow-derived mesenchymal stem cells (BMSCs) were purchased from American Type Culture Collection (ATCC, Manassas, VA). Mononuclear cells (2-3*×*10^5^ cells/cm^2^) were plated in a 100 mm culture dishes (Falcon Plastics, Los Angeles, CA, USA) with low glucose-Dulbecco’s modified Eagle’s medium (LG-DMEM; Gibco, Grand Island, NY, USA) containing 10% fetal bovine serum (FBS; Cat. No. 10099-141, Gibco, Auckland, New Zealand) and 1% penicillin/streptomycin (Gibco) and cultured at 37℃ in a 5% CO_2_ atmosphere. When the cultures approached 80% confluence, the cells were harvested by treatment with a trypsin/EDTA solution (Gibco) and replated at a density of 5*×*10^3^ cell/cm^2^ in culture plates. During culture, the medium was replaced with fresh medium every 3-4 days. Cells with passages 3 or 4 were used for the experiments treated with ginsenoside Rg3 (50 or 100 µg/mL, Cayman Chemical, Ann Arbor, MI, USA) or ethanol 0.25% (Millipore, Billerica, MA, USA) as a vehicle of Rg3. BAPTA-AM and EGTA was purchased from Sigma Aldrich (St. Louis, MO, USA) and Cayman Chemical, respectively.

**2. MTT assay**

Human BMSCs (2*×*10^3^ cells/well) were seeded onto 96-well plates (Falcon, Corning Inc., Durham, NC, USA) and cultured for 5 days with Rg3-containing medium in a dose-dependent manner. After 5 days culture, 0.5 mg of 3-(4,5-dimethylthiazol 2-yl)-2,5-diphenyltetrazolium formazan (MTT; Sigma, Saint Louis, MO, USA) dissolved in phosphate-buffered saline (PBS; Gibco) was added to each well and incubated at 37℃ for 4 h. MTT formazan was dissolved in 100 μL DMSO (Millipore) and each well was read at 570 nm on a microplate reader (Biotek, Winooski, VT, USA).

**3. RNA isolation and quantitative polymerase chain reaction (qPCR)**

Total RNA was isolated from human BMSCs using TRIzol reagent (Invitrogen, Carlsbad, CA, USA) according to the manufacturer’s protocol. RNA purity and concentration were determined using a microplate reader. cDNA was synthesized from total RNA (1 µg) by using the RT PreMix Kit (iNtRON Biotechnology, Seongnam, Gyeonggi-do, Korea). Transcript levels were measured by real-time PCR using sequence-specific primers. Amplification reactions contained SYBR green PCR Master Mix (Applied Biosystems, Warrington, UK) and were performed in the QuantStudio™ 6 Flex Real‐Time PCR System (Applied Biosystems) according to the manufacturer’s instructions. Sequence-specific primers are listed in supplementary figure 1. Data were analyzed by using QuantStudio 6 and 7 Flex Software (Applied Biosystems). The cycle threshold (Ct) values of the target genes were normalized to those of endogenous control gene (Peptidylprolyl isomerase A; PPIA). Relative changes were calculated by using the equation 2^-ΔΔCt^.

**4. Immunoblot analysis**

The cells were lysed using RIPA lysis buffer (Elpis-Biotech, Daejeon, Korea) with a protease inhibitor cocktail (Roche Diagnostics GmbH, Mannheim, Germany) and phosphatase inhibitor (Roche). The lysate protein concentrations were determined by using the BCA protein assay (iNtRON biotechnology). Equal amounts of protein (10 - 20 μg) in each sample were separated by 10-15% sodium dodecyl sulfate-polyacrylamide gel electrophoresis (SDS-PAGE) and transferred to polyvinylidene fluoride (PVDF; Millipore, Carrigtwohill, Ireland) membranes. The membranes were blocked with 5% skim milk in Tris-buffered saline containing 0.1% Tween 20 (TBST) for 1 h at room temperature and then incubated overnight with primary antibodies (1:1000) at 4℃. The primary antibodies were SOD2 (#13194), phospho-p70S6K (#9205), total-p70S6K (#9202), phospho-AMPK (#2535), total-AMPK (#2532), phospho-CREB (#sc-81486), total-CREB (#sc-240), MCU (#14997), VDAC (#4866) from Cell Signaling Technology (Danvers, MA); total OXPHOS antibody cocktail (#ab110413) from Abcam; GAPDH (#sc-47724) from Santa Cruz Biotechnology (Dallas, TX); Tfam kindly received from prof. Chanbae Park in Ajou University (Suwon, Korea); α-tubulin (#12G10) from Developmental Studies Hybridoma Bank.

All primary antibodies were diluted in TBST with 5% BSA. After washing three times in TBST, the membranes were incubated for 1 h in goat anti-mouse and goat anti-rabbit horseradish peroxidase-conjugated IgG secondary antibody (1:1000-3000 dilution; Invitrogen, Rockford, IL, USA). The membranes after washing several times were incubated in a developing solution (Enhanced chemiluminescence western blotting detection reagent; GE Healthcare, Buckinghamshire, UK) and signal was detected using Chemi-Doc System (Bio-rad, Hercules, CA, USA). Densitometric analyses of the Western blots were performed using Chemi-Doc software (Bio-rad).

**5. Senescence-associated β-galactosidase (SA-β-gal) staining**

Human BMSCs (5*×*10^3^ cells/well) were seeded in 24-well plates (Falcon) and cultured for 5 days with Rg3. SA-β-gal activity was assessed with a senescence β-gal staining kit (Cell Signaling Technologies, Danvers, MA, USA) according to the manufacturer’s instructions. The percentage of SA-β-gal positive cells was calculated by counting the number of blue stained cells under bright field illumination and then calculating the ratio of SA-β-gal staining positive cells (%) in each group.

**6. Osteogenic and Adipogenic Differentiation**

Osteogenic or adipogenic differentiation was determined by plating the human BMSCs (5*×*10^3^ cells/cm^2^) in 12-well plates (Falcon) and cultured for 5 days with Rg3. After reaching confluence, the human BMSCs were maintained for two or three weeks in either an osteogenic or adipogenic medium.

The osteogenic medium consists of low glucose (LG)-DMEM containing 10% FBS, 10 mM β-glycerophosphate (Sigma), 0.2 mM Ascorbic acid-2-phosphate (Sigma) and 100 nM dexamethasone (Sigma). The medium was replaced every 3-4 days and, after 14 days, Alizarin Red staining was performed to analyze the osteogenic differentiation of BMSCs. The cells were fixed in 4% formaldehyde (Duksan pure chemicals, Gyeonggido, Korea) for 30 min at room temperature, followed by 30 min in 2% Alizarin Red S staining solution (ScienCell, Carlsbad, CA, USA). To quantify the efficiency of osteogenic differentiation, the stained cells were eluted with DMSO and the optical density (OD) values were detected by spectrophotometer at a wavelength 540 nm.

The adipogenic medium consisted of high glucose (HG)-DMEM (Gibco) containing 10% FBS, 1 mM dexamethasone, 0.5 mM 1-methyl-3-isobutylxanthine (Sigma), 100 µM indomethacin (Sigma), and 10 µg/mL insulin (Sigma). After 21 days, Oil Red-O staining was performed to analyze the adipogenic differentiation of MSCs. The cells were fixed in 4% formaldehyde for 30 min at room temperature, followed by 20 min in 0.3% Oil Red O staining solution (Sigma). To quantify the efficiency of adipogenic differentiation, the stained cells were eluted with isopropanol and the OD values were detected by spectrophotometer at a wavelength 520 nm.

**7. Oxygen Consumption Rate (OCR) and Extracellular Acidification Rate (ECAR) measurement**

Human BMSCs plated on Seahorse 96 well plate (Agilent Technologies, Cedar Creek, TX, USA), and cultured for 5 days with Rg3. After culturing, cells were changed to pre-warmed XF DMEM medium (for OCR or ECAR measurement, containing 200 mM L-glutamate with or without 1M glucose and 100 mM pyruvate, then adjusted at pH 7.4; Agilent Technologies) 1 h prior to analyses and keeping cells at 37℃ without CO_2_. OCR and ECAR were measured in human BMSCs using the Seahorse XFe96 Analyzer (Agilent Technologies) as response to the addition of various chemicals. The cycles (3 times for 3 minutes) were run for every measurement and the XF Cell Mito Stress Test Kit (containing the following compounds: 2 μM oligomycin (ATP synthase inhibitor), 2 μM carbonyl cyanide-4-(trifluoromethoxy) phenylhydrazone (FCCP) (mitochondrial uncoupler), 0.5 μM rotenone (respiratory chain complex Ⅰ inhibitor) and antimycin A (complex Ⅲ inhibitor) ) and Glycolysis Stress Test Kit (including 10 mM glucose, 2 μM oligomycin and 50 mM 2-deoxy-D-glucose) (Agilent Technologies) were used. OCR and ECAR were normalized by protein amount determined by BCA protein assay.

**8. Measurement of cytosolic reactive oxygen species (ROS)**

The formation of cytosolic ROS was evaluated by the sensitive fluorescence probe 5-(and -6)-chloromethyl-2', 7'-dichlorodihydro-fluorescein diacetate, acetyl ester (CM-H_2_DCFDA; Invitrogen, Eugene, OR, USA). Human BMSCs were seeded in 100 mm dish, and cultured for 5 days with Rg3. After culturing and checking the cell number, the cell suspensions were centrifuged. The pellets were resuspended in PBS, and some were incubated as negative control. The others are treated with 5 µM DCFH_2_DA and incubated for 20 min at 37℃. In addition, 500 µM H_2_O_2_ was treated and incubated in part of DCFH_2_DA treatment for 10 minutes as a positive control for checking maximal ROS level. After incubation, fluorescence was measured at the excitation and emission wavelengths of 485 and 538 nm using a fluorescence-activated cell sorting (FACSAria III, BD Biosciences, USA). The level of ROS was normalized by cell number.

**9. Live-cell Ca^2+^ imaging**

Cells were seeded on poly-L-lysine coated 12 mm coverslip, and cultured for 5 days with Rg3. After culturing, cells were pre-incubated with 5 µM Fura-2/AM (Thermo Fisher Scientific) in Krebs-Ringer bicarbonate buffer (KRBB) containing, in mmol/L: 140 NaCl, 3.6 KCl, 2 NaHCO_3_, 0.5 NaH_2_PO_4_, 1.5 CaCl_2_, 0.5 MgCl_2_-7H_2_O and 10 HEPES, pH 7.4 plus 5.5 mmol/l glucose for 30 min at 37°C. Then, cells were transferred to a perfusion chamber on an inverted microscope (IX73, Olympus, Tokyo, Japan) and used of the 340/380 nm excitation by an illuminator (pe-340Fura; CoolLED, Andover, UK). Fluorescence images were captured at 510 nm with an intensified CCD camera (Prime-BSI; Teledyne Photometrics, Tucson, AZ, USA) and the ratio of fluorescence intensities (F340/F380) reflecting intracellular Ca^2+^ was analyzed by using MetaFluor 6.1 software (Molecular Devices, San Jose, CA, USA). At the end of each experiment, 10 µM ionomycin in KRBB was added to induce a maximal ratio. Based on the measured graph, the area under a curve (AUC) was calculated to identify the change in intracellular Ca^2+^.
